# The influence of training actives/inactives ratio on machine learning performance

**DOI:** 10.1186/1758-2946-5-S1-P30

**Published:** 2013-03-22

**Authors:** Rafał Kurczab, Sabina Smusz, Andrzej J Bojarski

**Affiliations:** 1 Department of Medicinal Chemistry, Institute of Pharmacology Polish Academy of Sciences, Kraków, 31-343, Poland; 2 Faculty of Chemistry, Jagiellonian University, Kraków, 30-060, Poland

## 

In drug discovery, machine learning is widely used to classify molecules as active or inactive against a particular target. The vast majority of these methods (supervised learning) needs a training set of objects (molecules) to develop a decision rule that can be used to classify new entities (the test set) into one of the two mentioned classes [1].

A lot of studies, searching an optimal learning parameters and their impact on classification effectiveness were performed [2,3]. Unfortunately, there is no data showing the influence of actives/inactives ratio, used to model training, on the efficiency of new active compounds identification. Therefore, the main goal of this study was to examine the impact of changing the number of inactives in the training set with fixed amount of actives. For a given ratio, the inactives were randomly selected from ZINC database (10-times to prevent an overestimations error). This concept was verified on three different protein targets (i.e. 5-HT_1A_, HIV-1 protease and matrix metalloproteinase) and a set of algorithms (SMO, Naïve Bayes, Ibk, J48 and Random Forest) implemented in WEKA package [4]. To compounds representation, two types of molecular fingerprints were used (MACCS and hashed fingerprint), to determine their possible impact on machine learning performance.
